# Technology as a Problem and Potential Solution in Teen Driving Safety

**DOI:** 10.1001/jamanetworkopen.2024.39281

**Published:** 2024-10-01

**Authors:** Cara Hamann, Jingzhen Yang

**Affiliations:** Department of Epidemiology, University of Iowa College of Public Health, Iowa City; University of Iowa Injury Prevention Research Center, Iowa City; Center for Injury Research and Policy, Abigail Wexner Research Institute at Nationwide Children’s Hospital, Columbus, Ohio; Department of Pediatrics, The Ohio State University, Columbus

Motor vehicle crashes (MVCs) continue to be the leading cause of death among adolescents in the US, making adolescent driving safety a top public health concern. In 2022, nearly 3000 adolescents between ages 13 and 19 years died due to MVCs and an additional 2.6 million had MVC-related injuries requiring emergency department visits.^1^ Limited experience and immaturity substantially contribute to the heightened risk among adolescents when they start driving. This risk is further amplified by distractions from the widespread use of electronic communications devices, such as cellphones and smartwatches. These distractions lead to drivers not focusing on the road, not keeping their hands on the wheel, and not concentrating on the task of driving.^1^

Cellphone use, in particular, handheld cellphone use, is associated with increased MVC risk.^1^ As such, cellphone use while driving is particularly problematic among adolescent drivers and strategies to prevent this dangerous behavior are urgently needed. McDonald et al^2^ addressed this issue with their recent work on the use of a smartphone telematics app and risky behavior detection algorithms to measure handheld cellphone use in a prospective sample of 119 newly licensed Pennsylvania adolescent drivers. The study examined the association between handheld cellphone use and kinematic risky driving (KRD) events, defined as a sum of increased gravitational *g* force events of hard braking (≤−0.40*g*) and rapid acceleration (≥0.35*g*) in a trip, among adolescent drivers. The findings suggest that handheld cellphone use occurred in a substantial proportion of trips and that trips with handheld cellphone use were associated with 2.62 times higher rates of KRD events.

The evidence generated by McDonald et al^2^ suggests the feasibility of an objective smartphone telematics app in monitoring risky adolescent driving that is very scalable beyond the scope of their study. It is ideal to use technology already present with adolescents—smartphones—as the tool to monitor driving, and the associated data are valuable in evaluating intervention efforts.

Prior research among adolescent drivers has largely aimed to increase overall road safety behaviors, not specifically focused on distraction. Centering on distraction can be challenging though, given there are many different types across the visual, manual, and cognitive spectrum, including handheld cellphone use, texting, and interactions with passengers. The strategies for monitoring and prevention of each of the distraction types often require different approaches.

Among prior work that has focused on measurement or prevention of cellphone use while driving, the evaluation approaches have largely relied on self-reports or driving simulation data, which may not accurately assess adolescent communication modes and driving scenarios or provide meaningful response options.^3^ For example, a study by Monroe et al^4^ evaluated changes in adolescent driving knowledge and self-reported behaviors before and after educational events to increase adolescent awareness of safe driving practices. Among their findings were reports of decreased texting while driving.

Additionally, technological advancements enable in-vehicle cameras to monitor high-risk driving events, but the evaluation process remains labor-intensive and time-consuming due to the large amounts of data collected and the need for manual coding, thus restricting our understanding of handheld cellphone use while driving.^2^ Similarly, studies based on roadway observations done from outside of the vehicle looking in may have biases in assessing driver characteristics and whether they are using a phone and, if so, how.^5^

The strength of the approach by McDonald et al^2^ is their use of a smartphone telematics app to directly detect cellphone use by adolescents and KRD events while driving, which overcomes the limitations of previous studies by eliminating the need for in-car monitoring and avoiding the bias typically found in self-reports, roadway observations, or simulations. Handheld cellphone use is considered one of the most dangerous types of distraction. The findings of McDonald et al^2^ suggest that smartphone telematics could serve as a potent tool for interventions aimed at enhancing safety by reducing handheld cellphone use.

It is well known that achievement of driving safety requires multilevel approaches that address not only driver behavior, but also the vehicle, roadway environment (ie, infrastructure), and social environment (eg, norms, policies).^6^ This multilevel concept aligns with the Safe System Approach, which has been adopted by the US Department of Transportation as their guiding paradigm for addressing road safety.^6^ The US Department of Transportation Safe System Approach has 5 objectives: safer people, safer roads, safer vehicles, safer speeds, and post-MVC care.^6^

Although just one piece of a safe system, driver behavior is an important factor to address, particularly among the most inexperienced and immature drivers as they are developing and shaping their safe driving habits. Multiple promising approaches exist to reduce distractive behaviors while driving among adolescents, including the smartphone app presented by McDonald et al.^2^ The current state of the science in the field of adolescent driving safety shows there is not a lack of possible intervention strategies for improving adolescent driver safety. However, the gaps that exist in the field primarily relate to consensus on best approaches, adoption and scale-up of evidence-based interventions, and gaps in policy, such as the strength of the electronic device use laws (eg, texting ban only vs universal hands-free law).

It is particularly important to understand how to promote uptake of the type of smartphone app presented by McDonald et al.^2^ It is likely that existing models, such as driver monitoring devices used by insurance companies,^7^ could be adapted by replacing the devices with smartphone apps tailored specifically for adolescent drivers. In addition, the smartphone app used by McDonald et al^2^ is capable of detecting and distinguishing handheld cellphone use over handsfree cellphone use (eg, playing music or using GPS through the cellphone), which allows for better understanding of the riskiest behaviors. Ultimately, McDonald et al^2^ contribute to the growing evidence suggesting that smartphone apps are a promising strategy to enhance driving safety among adolescents, benefiting not only adolescents themselves, but all road users.
